# Chernobyl Cancer Studies with Overseas Control: High Grade vs. Late Detection

**DOI:** 10.5146/tjpath.2021.01526

**Published:** 2022-09-15

**Authors:** Sergei Jargın

**Affiliations:** Department of Pathological Anatomy, Peoples’ Friendship University of Russia, Moscow, Russian Federation

**Dear Editor,**

This is an addition to the review published in the Turkish Journal of Pathology (1), commenting on the series of studies (2–7), in particular, the last one making a comparison of clear-cell renal carcinoma (RC) tissue specimens from Ukraine with those from Colombia and Spain (7). Thyroid cancer (TC) is discussed by analogy. RCs from Ukraine tended to be higher-grade than those from Spain and Colombia (2–7); among others, they displayed a sarcomatoid i.e. poorly differentiated pattern more frequently: 62 from 236 (26.3%) of Ukrainian vs. 11 from 112 (9.8%) of Spanish cases (p<0.001) (2). The statistically significant difference was confirmed in the later work (4). In the recent study, the microvessel density in RC tissue from patients residing both in “highly” and “low contaminated areas of Ukraine” (7) was higher than that in RC from Spain and Colombia (p<0.01). The difference between the two Ukrainian groups was statistically insignificant. The increased angiogenesis was associated with a higher expression of VEGF (7). It was assumed that the exposure to ionizing radiation leads to an increase in the microvessel density, which in turn is associated with a higher grade of RC (6,7). In this connection, the following citations should be commented: “The dramatic increase of aggressivity and proliferative activity” was found in RC from Ukraine, while “the majority of the high grade tumors occurred in the Ukrainian (rather than in the Spanish) groups” (2). These differences can be explained by the earlier cancer detection on average in Spain and discovery by the screening of advanced cases in Ukraine (8).

Annual average doses from the natural radiation background should be indicated in the studies comparing patients from different countries; otherwise doses in controls may turn out to be not significantly different from those in the “exposed” group. No doses are given in the articles (2–7). Individual effective doses from the natural background are generally expected to range from 1.0 to 10 mSv/year; some national averages exceed 10 mSv/year (9,10). The average for the Russian Federation is estimated to be 3.35 mSv/year; the highest background among federal subjects is in the Altai Republic - 8.83 mSv/year (11). “The six million residents of the areas of the former Soviet Union deemed contaminated received average effective doses for the period 1986-2005 of about 9 mSv” (12). The average annual individual dose from the natural background radiation in Spain is around 5 mSv (13). Carcinogenesis has never been proven for the above dose levels.

Thyroid doses after the accident were higher because of the radioiodine accumulation. There has been no convincing evidence of cause-effect relationships between radiation exposures from the Chernobyl fallout and the incidence increase of cancers other than TC (12). Mechanisms of TC detection rate elevation included the screening effect and improved medical surveillance (12). As discussed previously, the registered incidence of pediatric TC in the former Soviet Union prior to the Chernobyl accident had been lower than in other developed countries, which indicates that there were undiagnosed cases in the population (1,14). Apparently, some neglected cancers, detected by the screening or self-reporting (due to the increased public awareness after the accident), were interpreted as rapidly growing radiogenic malignancies. Moreover, non-exposed patients were probably counted among exposed ones. Some people strived for the recognition as Chernobyl victims to gain access to health care provisions. Cases from non-contaminated areas must have been more advanced on average as there was no extensive screening there; more details and references have been provided previously (1,14). The same probably pertains to some extent also to RC.

Considering the above, certain characteristics of RCs from Ukraine vs. those from Spain and Colombia need an interpretation e.g. the absence of significant differences in the expression of ubiquitin (5). Assuming that RCs from Ukraine were more advanced than Spanish cases on average, the data indicate that ubiquitin is not associated with the progression and de-differentiation of RC. On the contrary, VEGF was found more frequently in RCs from Ukraine than in those from Spain and Colombia (7). The “study demonstrated a close relationship between VEGF expression and the stage of clear-cell RC” (7). Apparently, the microvessel density and VEGF expression correlate with the tumor de-differentiation and hence with disease duration. The same is probably true for other markers, where significant differences between the Spanish and Ukrainian RCs were found e.g. the transcriptional nuclear factor kappa B (NF-kappa-B), and its p50 and especially p65 subunits (4). The >10% cell positivity for p50 was found in 25 from 59 (42.4%) of Ukrainian vs. 4 from 19 (21.1%) of Spanish cases; the >50% p65 positivity was found, correspondingly, in 18 from 59 (30.1%) vs. 1 from 19 (5.3%) of the cases (p<0.05) (4). Accordingly, NF-kappa-B activation is discussed in the literature as a probable marker of cancer progression.

The suppositions about enhanced aggressiveness of tumors from radiocontaminated areas might be conducive to overtreatment. Surgeons may decide to perform nephrectomy instead of a kidney-preserving procedure if they learn that cancers from contaminated areas are more aggressive than usual, where the peritumoral renal parenchyma harbors “proliferative atypical nephropathy with tubular epithelial nuclear atypia and carcinoma in situ” (3). The potential overtreatment of post-Chernobyl thyroid and urinary bladder lesions has been discussed elsewhere (14,15).
